# Effect of sodium ascorbate on the bond strength of all-in-one adhesive systems to NaOCl-treated dentin

**DOI:** 10.4317/jced.52512

**Published:** 2015-12-01

**Authors:** Mohammad-Esmaeel Ebrahimi-Chaharom, Soodabeh Kimyai, Narmin Mohammadi, Parnian-Alizadeh Oskoee, Mehdi Daneshpuy, Mahmoud Bahari

**Affiliations:** 1 Dental and Periodontal Research Center, Tabriz University of Medical Sciences, Tabriz, Iran; 2 Associate Professor, Department of Operative Dentistry, Faculty of Dentistry, Tabriz University of Medical Sciences, Tabriz, Iran; 3 Professor, Department of Operative Dentistry, Faculty of Dentistry, Tabriz University of Medical Sciences, Tabriz, Iran; 4 Assistant Professor, Department of Operative Dentistry, Faculty of Dentistry, Tabriz University of Medical Sciences, Tabriz, Iran

## Abstract

**Background:**

Ascorbic acid and its salts are low-toxicity products, which are routinely used in food industries as antioxidants. The aim of the present study was to evaluate the effect of 10% sodium ascorbate on the bond strength of two all-in-one adhesive systems to NaOCl-treated dentin.

**Material and Methods:**

After exposing the dentin on the facial surface of 90 sound human premolars and mounting in an acrylic resin mold, the exposed dentin surfaces were polished with 600-grit SiC paper under running water. Then the samples were randomly divided into 6 groups of 15. Groups 1 and 4 were the controls, in which no surface preparation was carried out. In groups 2 and 5 the dentin surfaces were treated with 5.25% NaOCl alone for 10 minutes and in groups 3 and 6 with 5.25% NaOCl for 10 minutes followed by 10% sodium ascorbate for 10 minutes. Then composite resin cylinders, measuring 2 mm in diameter and 2 mm in height, were bonded on the dentin surfaces in groups 1, 2 and 3 with Clearfil S3 Bond and in groups 4, 5 and 6 with Adper Easy One adhesive systems according to manufacturers’ instructions. The samples were stored in distilled water for 24 hours at 37°C and then thermocycled. Finally, the samples underwent shear bond strength test in a universal testing machine at a strain rate of 1 mm/min. Data were analyzed with two-way ANOVA and post hoc Tukey tests at α=0.05.

**Results:**

The differences between groups 1 and 2 (*P*=0.01), 1 and 5 (*P*=0.003). 1 and 6 (*P*=0.03) and 4 and 5 (*P*=0.03) were statistically significant. Two-by-two comparisons did not reveal any significant difference between other groups (*P*>0.05).

**Conclusions:**

Use of 10% sodium ascorbate for 10 minutes restored the decreased bond strength of the adhesive systems to that of the control groups.

** Key words:**Sodium ascorbate, adhesive systems, all-in-one, bond strength, sodium hypochlorite.

## Introduction

Endodontically treated teeth lose a large proportion of their structure due to trauma and caries and during endodontic treatment. As a result, they have low physical properties, fracture resistance and esthetic appearance (1). Restoration of endodontically treated teeth is a very important step to achieve clinical success and to restore function and esthetic (2). Some of the advantages of composite resin restorations are their ability to bond to dentin, increase resistance to fracture of the remaining tooth structure, increase retention of the restoration, decrease marginal microleakage and enhance the esthetic appearance (3).

At present, the adhesive systems are generally divided into etch-and-rinse and self-etch systems, with differences in their mechanism of adhesion to tooth structures and in their bond strength. All-in-one adhesives are a member of the self-etch adhesive system in which the etchant, the primer and the bonding agent have been incorporated in one bottle; therefore, they do not require separate etching, rinsing and drying steps, resulting in saving time (4).

Sodium hypochlorite (NaOCl) solution is the most commonly used root canal irrigation solution which has antibacterial activity and can dissolve organic tissues, too (5). Although the use of NaOCl has some advantages, it has some disadvantages, too, the most important of which include remaining of the irrigation solution or its derivatives (nascent oxygen) in the root canal and its negative effect on the polymerization of the adhesive system, finally resulting in a decrease in bond strength. Several studies have shown the effect of NaOCl on decreasing the bond strength of etch-and-rinse (6,7) and two-step self-etch adhesive systems (8,9).

On the other hand, the decrease in bond strength due to the use of NaOCl can be reversed with the use of some antioxidant agents such as sodium ascorbate (10), green tea extract (11), proanthocyanidins (12) and rosemarinic acid (8). The antioxidative agents can increase the bond strength and return it to the normal level by breaking the free radical chains, metal chelating, repulsion of free radicals and reacting with secondary products of NaOCl to neutralize them (10). Several studies have shown the capacity of 10% sodium ascorbate to restore the decreased bond strength of etch-and-rinse adhesive systems (13,14). In contrast, some studies demonstrated that the application of sodium ascorbate solution did not significantly increase the compromised bonding of two-step self-etch adhesive systems to NaOCl treated dentin (8,15). So, The aim of the present study was to evaluate the effect of 10% sodium ascorbate on the shear bond strength of two all-in-one adhesive systems on the NaOCl-treated dentin.

## Material and Methods

Ninety sound human premolar teeth with closed apices, extracted for orthodontic reasons from subjects 15-25 years of age, were selected and used for the purpose of this in vitro study. The teeth were placed in 0.5% chloramines T solution (Merck, Hamburg, Germany) at 4°C immediately after extraction. One week before the laboratory procedures the teeth were cleaned of all the calculi and soft tissue remnants and then stored in distilled water. The teeth were cut by a diamond disk (Diament Gmbh, D82, Goerzallce 307, 14167 Berlin, Germany) in a high-speed handpiece under air and water coolant in a transverse plane perpendicular to the long axes of the teeth. After elimination of enamel, the dentin was exposed. A 10-mL syringe was used to mount the samples in self-cured acrylic resin with the cut dentin surface flush with the acrylic resin surface. The exposed dentin surfaces were polished with 600-grit silicon carbide paper under running water to achieve a smooth surface with a homogeneous smear layer. Then the samples were randomly divided into 6 groups using the Randlist software program as follows.

In groups 1 and 4 (S1 and E1, respectively) no preparation was carried out.

In groups 2 and 5 (S2 and E2, respectively) the samples were exposed to 5.25% NaOCl (Merck Darmstadt, Germany) for 10 minutes.

In groups 3 and 6 (S3 and E3, respectively) the samples were exposed to 5.25% NaOCl for 10 minutes and then rinsed with 10% sodium ascorbate (Fluka, Buchs, Switzerland) for 10 minutes.

In groups 1 to 3 (S1, S2 and S3) Clearfil S3 Bond (Kuraray Medical Inc., Japan) and in groups 4 to 6 (E1, E2 and E3) Adper Easy One (3M ESPE, St. Paul, MN, USA) adhesive systems were used according to manufactures’ instructions. Z100 (3M Co., St. Paul, MN, USA) composite resin was used to construct composite resin cylinders in transparent plastic molds measuring 2 mm in diameter and 2 mm in height. To this end, the transparent cylinders were filled with the A1 shade of composite resin and placed on the prepared dentin surface fixed by pincers. A piece of celluloid matrix band was placed on it and pressed by finger. Then extra composite resin material was removed using a dental explorer. The composite resin cylinders were light-cured for 40 seconds using Astralis 7 (Ivoclar Vivadent, FL 9494 Schaan) light-curing unit at a light intensity of 400 mW/cm3 with the light-conducting tip touching and perpendicular to the surface from 2 directions (20 seconds from each direction). The composite resin cylindrical samples were stored in distilled water for 24 hours at 37°C and then underwent a thermocycling procedure consisting of 500 rounds at 5±2/55±°C. Finally, the samples underwent a shearing bond test in a universal testing machine (Hounsfield Test Equipment, H5k-S Model, Surray, England) at a strain rate of 1 mm/min by placing the chisel-shaped blade of the test equipment at dentin‒composite resin interface.

The mean and standard deviation for each group were calculated and analyzed using two-way ANOVA and post hoc Tukey tests. Statistical significance was defined at *P*<0.05. Fracture patterns were determined by evaluating the samples under a stereomicroscope (Nikon, Tokyo, Japan) at a magnification of ×40. The fracture patterns were classified as adhesive, cohesive and mixed and the fracture pattern frequencies were reported as percentages in the study groups.

## Results

 Table 1 presents the means, standard deviations and standard errors of shear bond strength values in all the study groups.

**Table 1:**
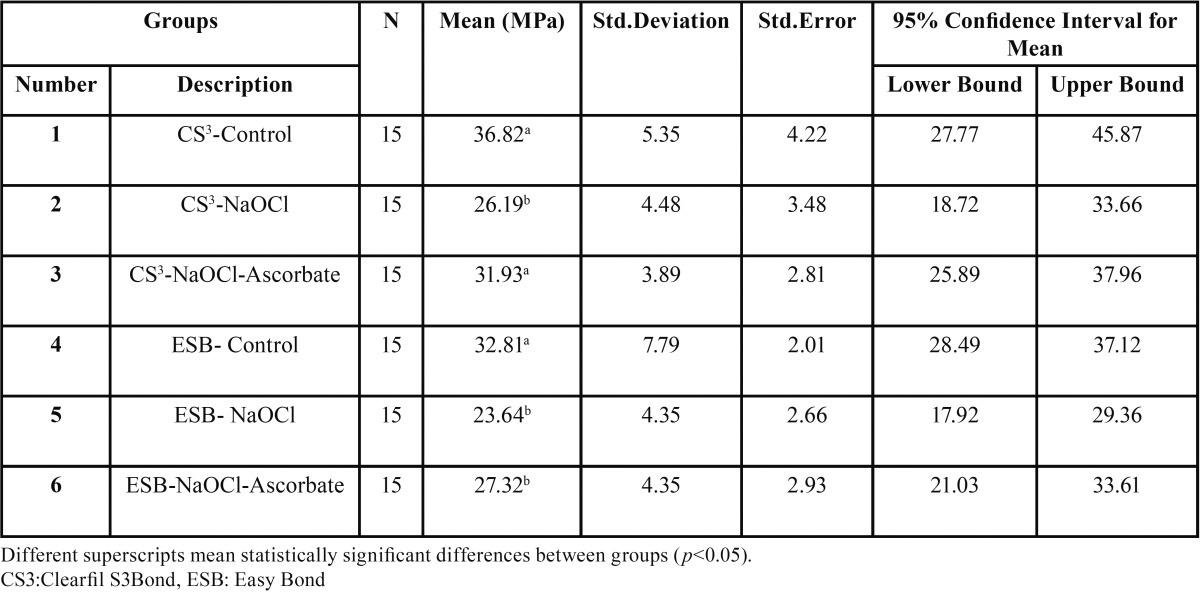
The means, standard deviations and standard errors of shear bond strength values in the study groups.

The results of one-way ANOVA showed significant differences in shear bond strength values between the different study groups (*P*=0.03). Two-by-two comparisons of the groups with post hoc Tukey test showed that differences between groups 1 and 2 (*P*=0.01), 1 and 5 (*P*=0.003), 1 and 6 (*P*=0.03), and 4 and 5 (*P*=0.03) were significant. There were no significant differences between the other groups (*P*>0.05). In other words, NaOCl treatment decreased bond strength of both adhesive systems significantly in comparison to their controls. Furthermore, application of 10% sodium ascorbate for 10 minutes on the dentin treated with 5.25% NaOCl increased the bond strength of both all-in-one adhesive systems to values comparable to those in the corresponding control group.

## Discussion

Sodium hypochlorite is a well-known nonspecific proteolytic agent, capable of eliminating organic materials and magnesium and carbonate ions (16). It is used as a deproteinizing agent on dentin (17). Several studies have evaluated the role of NaOCl in dentin permeability and adhesion to dentin (18,19). Depending on the study procedure and methodology or the specific chemical structure of the adhesive system used, use of NaOCl after etching results in an increase or decrease in bond strength (19-21). Tanaka *et al.* (22) reported that there is an increase in bond strength to dentin with an increase in the concentration of NaOCl until it reaches a plateau at a concentration of 10% with an application time of 60 seconds.

In the present study the dentin bond strength of all-in-one self-etch adhesive systems was evaluated after application of 5.25% NaOCl for 10 minutes. The results showed a significant decrease in the bond strengths of the two adhesive systems after application of NaOCl compared to the control group. Several researchers have reported a decrease in bond strength between the compo-site resin and dentin with the use of etch-and-rinse (6,7) and two-step self-etch adhesive systems (8,9) after the application of NaOCl.

In a study by Perdigao *et al.* (17) treating dentin collagen fibers with NaOCl decreased the bond strength of two single-bottle adhesive systems despite deep penetration of the adhesive. They argued that the demineralization depth of the dentin might not have an important role in adhesion to dentin; rather, the quality and integration with the available dentin during resin penetration might have a more important role. A decrease in the bond strength of etch-and-rinse adhesive systems after etching might be attributed to different mechanisms, including the relative dissolution of collagen at intertubular areas (23), instability of collagen molecules (24), shrinkage of dentin (25) and a change in crystallization degree of dentin hydroxyapatite after application of Na-OCl (17,25).

The main difference between the self-etch adhesive systems and their previous generation is the fact that the self-etch systems do not need a separate etching step, and the etching solution and the primer have been incorporated in one bottle; therefore, etching and priming steps are carried out simultaneously (4). As a result, it is believed that a decrease in the bond strength of self-etch adhesive systems can be attributed to the remnants and by products of NaOCl, which have a negative effect on the polymerization of the adhesive systems. Treating the dentin with NaOCl might lead to biologic oxidation (26). In other words, the remnants of free radicals resulting from the oxidative effect of NaOCl on vinyl free radicals produced after activation of the adhesive systems compete with light and result in incomplete polymerization due to the premature termination of the chain (27,28). A decrease in bond strength due to NaOCl might also be attributed to the production of oxygen due to the disintegration of NaOCl to NaCl and oxygen. The oxygen released due to this process is a strong factor, preventing polymerization of the adhesive agents (13).

In addition, the present study showed that use of 10% sodium ascorbate for 10 minutes on the dentin treated with 5.25% NaOCl significantly increased the bond strength of both all-in-one adhesive systems. In previous studies, too, the positive and significant effect of the use of 10% sodium ascorbate has been shown in increasing the bond strength of etch-and-rinse (6,7) and self-adhesive cements (29) to dentin treated with NaOCl. Based on the results of a study by Vongphan *et al.* (14) the effect of sodium ascorbate on increasing the bond strength was predominantly attributed to chemical reactions. In other words, the product resulting from a chemical reaction between sodium ascorbate and NaOCl was responsible for an increase in bond strength because in that study repetition of the rinsing procedure with water after application of sodium ascorbate resulted in a significant decrease in bond strength (14). Since ascorbate acid and its sodium salt are potential anti-oxidants, it is probable that sodium ascorbate can change oxidative agents through a redox reaction (6). In other words, sodium ascorbate can promote the polymerization reaction of free radicals of the adhesive agent without premature termination and reverses the disrupted bonding to NaOCl-treated dentin (13).

Furthermore, In the present study, the results achieved in the sodium ascorbate group were similar to those achieved in the corresponding control group. Vitamin C and its salts, such as sodium ascorbate, are non-toxic and are used in food industries and it appears their application on dentin has no reverse biologic effects (30). Future studies should focus on determining the minimum time and concentrations necessary for the application of sodium ascorbate, with preservation of its positive effects, on evaluation of an increase in the durability of the bond with the use of different adhesive systems and on evaluation of microleakage.
